# Human Voltage-Gated Proton Channel Hv1: A New Potential Biomarker for Diagnosis and Prognosis of Colorectal Cancer

**DOI:** 10.1371/journal.pone.0070550

**Published:** 2013-08-05

**Authors:** Yifan Wang, Xingye Wu, Qiang Li, Shangrong Zhang, Shu Jie Li

**Affiliations:** 1 Department of Biophysics, School of Physics Science, Nankai University, Tianjin, China; Department of Pathology, Tonghua Center Hospital, Tonghua, China; The University of Kansas Medical center, United States of America

## Abstract

Solid tumors exist in a hypoxic microenvironment, and possess high-glycolytic metabolites. To avoid the acidosis, tumor cells must exhibit a dynamic cytosolic pH regulation mechanism(s). The voltage-gated proton channel Hv1 mediates NADPH oxidase function by compensating cellular loss of electrons with protons. Here, we showed for the first time, that Hv1 expression is increased in colorectal tumor tissues and cell lines, associated with poor prognosis. Immunohistochemistry showed that Hv1 is strongly expressed in adenocarcinomas but not or lowly expressed in normal colorectal or hyperplastic polyps. Hv1 expression in colorectal cancer is significantly associated with the tumor size, tumor classification, lymph node status, clinical stage and p53 status. High Hv1 expression is associated significantly with shorter overall and recurrence-free survival. Furthermore, real-time RT-PCR and immunocytochemistry showed that Hv1 is highly expressed in colorectal cancer cell lines, SW620, HT29, LS174T and Colo205, but not in SW480. Inhibitions of Hv1 expression and activity in the highly metastatic SW620 cells by small interfering RNA (siRNA) and Zn^2+^ respectively, markedly decrease the cell invasion and migration, restraint proton extrusion and the intracellular pH recovery. Our results suggest that Hv1 may be used as a potential biomarker for diagnosis and prognosis of colorectal carcinoma, and a potential target for anticancer drugs in colorectal cancer therapy.

## Introduction

The voltage-gated proton channel Hv1 was identified using bioinformatics searches based on known cation channels, which is mainly expressed in immune cells such as macrophages, neutrophils, and eosinophils [1], [2]. Hv1 in mammalian phagocytes was proposed to be responsible for the proton-transporting pathway, which regulates intracellular pH during oxygen consumption associated with phagocytosis, called “respiratory burst” [3], [4]. Hv1 is activated by depolarization and intracellular acidification, whose activity maintains intracellular pH neutral to keep reactive oxygen species (ROS) generation [5], [6]. Hv1 not only regulates pH in cytoplasm, but can also provide protons in the phagosome, a closed membrane compartment for killing and digestion of a pathogen [3]. Hv1 is extremely selective for H^+^, with no detectable permeability to other cations [7], [8]. The voltage activation relationship of Hv1 depends strongly on both the intracellular pH (pH_i_) and extracellular pH (pH_o_). Increasing pH_o_ or lowering pH_i_ promotes H^+^ channel opening by shifting the activation threshold to more negative potentials [3]. Furthermore, Hv1 current is inhibited by submillimolar concentrations of Zn^2+^ and Cd^2+^ and other divalent cations [9].

Hv1 contains three predicted domains: N-terminal acid and proline-rich domain, transmembrane voltage-sensor domain (VSD), and C-terminal domain. Voltage-gated K^+^ channels are comprised of four subunits, each of which has a pore domain and a VSD. The four pore domains come together to form one single central pore, and four peripheral VSDs control the gate of the pore [10]. In contrast to the voltage-gated K^+^ channels, the Hv1 contains a VSD but lacks the pore domain. Recent studies showed that Hv1 functions as a dimer in which the intracellular C-terminal domain is responsible for the dimeric architecture of the protein, and each subunit contains its own proton-transporting pathway [11]–[14]. The intracellular C-terminal domain of Hv1 forms a dimer via a parallel *α*-helical coiled-coil and is essential for the protein localization [14].

Tumor cells often exist in a hypoxic microenvironment, and possess high-glycolytic activity and produce acidic metabolites [15], [16]. To avoid the acidosis resulting from reducing in cytosolic pH, tumor cells must extrude excessing cytosolic protons to maintain cytosolic pH, which results in acidic tumor microenvironment. The hypoxic and acidic tumor microenvironment plays a key role in cancer development, progression, and metastasis [15]. Our previous work showed that Hv1 is specifically expressed in highly metastatic human breast tumor tissues and cell lines, and promotes breast cancer cell progression and metastasis, through regulating breast cancer cell intracelular pH [17], [18]. In the present study, we investigated the expression of Hv1 in colorectal tumor tissues and cell lines and its potential association with clinicopathological features and post-resectional survival. Inhibitions of Hv1 expression and activity in the highly metastatic colorectal cancer cells markedly decrease the cell invasion and migration, restraint proton extrusion. Our results suggest that Hv1 over-expression may be used as an independent biomarker for the prognosis and diagnosis of patients with colorectal cancer.

## Materials and Methods

### Ethics Statement

All of the procedures were done in accordance with the Declaration of Helsinki and relevant policies in China. We obtained the written informed consent from all participants involved in our study. The study obtained ethics approval for our study from the ethics committee of Tonghua Center Hospital.

### Patients and samples

Colorectal cancer tissue samples were obtained from patients who underwent routine curative surgery at the Department of Surgery, Tonghua Center Hospital between 2001 and 2007. The patients were not pretreated with radiotherapy or chemotherapy prior to surgery. 139 colorectal cancer tissues and paired adjacent non-tumor colorectal tissues were fixed in 10% formalin and embedded in paraffin for immunohistochemical analysis. The clinicopathological features of these patients were shown in Table 1. In addition, to verify the expression of Hv1 in premalignant dysplastic lesions, 10 normal colorectal, 18 hyperplastic polyp and 20 adenoma tissues were also examined using immunohistochemistry. Each patient's clinical status was classified according to the pathologic tumor grade, tumor size, lymph node status. Tumor differentiation was graded by the Edmondson grading system. This study was approved by the Ethics Committee of Tonghua Center Hospital, and informed consent was obtained from each patient.

**Table 1:** Clinicopathological parameters.

Characteristics		No. of patients (%), n = 139
Gender	Male	68(48.9)
	Female	71(51.1)
Age (yr) median 62.4, range 36–76	>62	79(56.8)
	≤62	60(43.2)
Primary site	Left colon	29(20.9)
	Right colon	43(30.9)
	Rectum	67(48.2)
Tumor size (cm)	<5	72(51.8)
	≥5	67(48.2)
Tumor classification	T1	5(3.6)
	T2	40(28.8)
	T3	85(61.1)
	T4	9(6.5)
Lymph node status	N0	80(57.5)
	N1	44(31.7)
	N2	15(10.8)
Distance metastasis	M0	132(95.0)
	M1	7(5)
Clinical stage	I/II	78(56.1)
	III/IV	61(43.9)
Differentiation	Well	61(43.9)
	Moderately	58(41.7)
	Poorly	20(14.4)

### Generation of an anti-Hv1 polyclonal antibody

An anti-Hv1 polyclonal antibody was generated against the carboxyl terminal domain of Hv1 (residues 221–273 of Hv1, KTRSERQLLRLKQMNVQLAAKIQHLEFSCSEKEQEIERLNKLLRQHGLLGEVN). The protein was purified to homogeneity after expression in *Escherichia coli* [19]. The purified protein was injected into mice and the anti-Hv1 polyclonal antibody was purified by rProtein A Sepharose (GE, Healthcare) column. HRP-conjugated goat anti-mouse IgGs were purchased from Jackson ImmunoResearch Laboratories, Inc. (West Grove, PA).

### Expression vector and transfection

Hv1 cDNA was cloned into pEGFP-N1 (Clontech) to create Hv1 expression plasmid pHv1-EGFP, fused with the enhanced green fluorescent protein (EGFP) moiety attached to the C-terminus of Hv1 [14]. 293 T cells were cultured in DMEM (Dulbecco's modified Eagle's medium; GIBCO) with 10% fetal bovine serum plus antibiotics (100 units/ml penicillin and 100 g/ml streptomycin, GIBCO) in a 5% CO_2_ incubator at 37°C. Cells grown on glass coverslips at 50–70% confluence in a six-well plate were transiently transfected with pHv1-EGFP plasmid by using lipofectamine 2000 (Invitrogen) following the manufacturer's protocol. Cells were used for experiments 36–48 h after transfection.

### Western blotting

Western blotting was performed with an anti–Hv1 polyclonal antibody (1 mg/ml) as described above with a final dilution of 1 1000. The denatured proteins were separated by 12.5% SDS-PAGE and then transferred to a PVDF membrane (GE, Healthcare) by wetting electroblotting devices. Nonspecific protein absorption was prevented using 5% (w/v) skim milk in phosphate-buffered saline containing 0.1% Tween 20 (PBS-T) for 1 h. Primary antibody incubation in PBS-T was performed for 1 h at room temperature. The HRP-coupled anti-mouse secondary antibody was used at a final dilution of 1 15,000 in PBS-T, and HRP was revealed with a chemiluminescent detection system (Millipore).

### Immunohistochemistry

Histological diagnoses of tumourous and non-tumourous formalin-fixed and paraffin-embedded tissues were confirmed in haematoxylin and eosin-stained sections. Immunohistochemistry was performed with an anti-Hv1 polyclonal antibody. The anti-Hv1 antibody (1.0 mg/ml) was diluted 100-fold with PBST (phosphate-buffered saline containing 0.1% tween-20) containing 1% (w/v) BSA. The paraffin-embedded sections filled with 10 mM ethylenediaminetetracetic acid (EDTA) buffer (pH 8.0) were heated in a microwave oven for 12 min. After cooling, the sections were treated with 0.5% Triton X-100 in PBS for 10 min, exposed to 3% (v/v) hydrogen peroxide (H_2_O_2_) for 10 min to inhibit endogenous peroxidase activity. Subsequently, the sections were incubated in PBST containing 5% fetal bovine serum and 2% BSA for 30 min to reduce nonspecific binding. Incubation with primary antibody was performed overnight at 4°C in a humidified chamber. HRP-coupled anti-mouse secondary antibody was used at a final dilution of 1 400 for 1 h. Finally, the visualization signal was developed with diaminobenzidine (DAB) and the slides were counterstained in hematoxylin. Negative control was performed to treat with non-immune mouse serum as the primary antibody instead of anti-Hv1 antibody.

Stained sections were evaluated in a blinded manner without prior knowledge of the clinical information using the German immunoreactive score, Immuno-Reactive-Score (IRS). Briefly, the IRS assigns sub-scores for immunoreactive distribution (0–4) and intensity (0-3), then multiplies them to yield the IRS score. The percent positivity was scored as “0” (<5%), “1” (5–25%), “2” (25–50%), “3” (50–75%), “4” (>75%). The staining intensity was score according to the area of Hv1-positive staining cells as “0, −” (negative, <5%), “1, +” (weakly positive, 5–25%), “2, ++” (positive, 25–50%), and “3, +++” (strongly positive, >50%). The final Hv1 expression score was calculated from the values of percent positivity score and staining intensity score, which was ranged from 0 to 12. We estimated IRS by averaging the values in eight fields at ×500 magnification for each specimen. Hv1 expression levels were defined as follows: low expression (score ≤3) and high expression (score >3). Immunohistochemical analysis and scoring were performed by two independent experienced pathologists.

### Cell culture

The human colorectal cancer cell lines SW620, HT29, LS174T, Colo205 and SW480 were cultivated at 37°C in an atmosphere of 95% air and 5% CO_2_ with DMEM supplemented with 10% fetal bovine serum (FBS), 100 U/ml penicillin, 100 µg/ml streptomycin, and 20 mM L-glutamine.

### Immunocytochemistry

SW620, HT29, LS174T, Colo205 and SW480 cells grown on glass coverslips at confluence in six-well tissue culture plates were fixed with 4% (w/v) paraformaldehyde in PBS at room temperature for 30 min, washed in PBS, treated with 0.5% Triton X-100 in PBS for 20 min, exposed to 3% (v/v) hydrogen peroxide (H_2_O_2_) for 15 min, and blocked with 5% fetal bovine serum and 2% BSA in PBST for 20 min at room temperature. The blocked coverslips were incubated with the anti-Hv1 antibody (1.0 mg/ml) at a dilution of 1 200 with PBST containing 1% (w/v) BSA at 37°C for 1 h. After washing in PBS for three times, the coverslips were further incubated with HRP-coupled anti-mouse secondary antibody at a final dilution of 1 400 for 1 h. Signals were visualized by the HRP/DAB system. The cell nuclei were stained with hematoxylin.

### Quantitative real-time PCR

The mRNA expression levels of Hv1 in SW620, HT29, LS174T, Colo205 and SW480 cells, were evaluated by quantitative real-time PCR using ABI PRISM 7000 Sequence Detection System (Applied Biosystems, Foster City, CA). Total RNAs were extracted using RNAiso Reagent (Takara), and reverse-transcribed by MMLV super transcriptase (Takara). Real-time quantitative reverse transcription PCR was performed with SYBR PrimeScriptTM RT-PCR Kit (Takara) according to the manufacturer's instructions. The primers were as follows: Hv1, 5′-CAGGTCATCATCATCTGCTTG-3′ (forward) and 5′-CCGTTCTGAACGTGTCTTAAC-3′ (Reverse); GAPDH, 5′-CCAAGGTCATCCATGACAAC-3′ (forward) and 5′-AGAGGCAGGGATGATGTTCT-3′ (reverse). PCR thermal condition used was: 94°C for 30 s; annealing, 52°C for 30 s; extension, 72°C for 30 s. Relative mRNA expression levels of proteins were calculated according to the equation: 2^−ΔΔCT^, in which ΔΔCT = (CT_Hv1_ - CT_GAPDH_) - (CT_Hv1_ - CT_GAPDH_)_SW620_. All experiments were performed in triplicate.

### Immunofluorescence

SW620 and SW480 cells grown on glass coverslips were fixed with 4% (v/v) paraformaldehyde in phosphate-buffered saline (PBS) at room temperature for 30 min, washed in PBS, treated with 0.5% Triton X-100 in PBS for 20 min, and blocked with 5% fetal bovine serum and 2% BSA in PBST for 1 h. The blocked coverslips were incubated with anti-Hv1 antibody (1.0 mg/ml) at a dilution of 1 200 in 2% BSA at 4°C overnight. After washing with PBS for 5 min for four times, the coverslips were further incubated for 1 h at room temperature with a FITC-conjugated goat anti-mouse IgG at a dilution of 1 400 in 2% BSA, followed by another washing as described above. Confocal images of FITC fluorescence of SW620 and SW480 cells were recorded on a Leica TCS SP5 confocal microscopy (LEICA, Germany) with the FITC-filter set for Hv1 and the DAPI filter set for the nuclear DAPI dye. The images were later processed by Adobe Photoshop software.

### Suppressing Hv1 mRNA expression

The sequence of the siRNA targeting the Hv1 gene was 5′-CTACAAGAAATGGGAGAAT-3′, and the random sense sequence was 5′-TTCTCCGAACGTGTCACGT-3′, both of which were obtained from Ribobio (Guangzhou, China) [17]. SW620 cells grown at confluence were passed in 6-well plates at 30% confluence and incubated overnight, then transfected with the siRNA and the negative control, respectively, using lipofectamine 2000 (Invitrogen), according to the manufacturer's protocol. The final concentration of siRNA was 100 nM. Silencing was examined 48 h after transfection. The efficiency of siRNA in suppressing Hv1 expression was determined by quantitative real-time PCR, immunocytochemistry and western blotting using anti-Hv1 antibody as described above.

### Migration kinetics

To examine the effect of Hv1 on the migratory ability of colorectal cancer cells, migrations of SW620, SW480, and SW620 cells down-regulated Hv1 expression and inhibited Hv1 activity by siRNA and Zn^2+^ respectively, were assessed in wounded monolayer model. To down-regulate Hv1 expression, SW620 cells were grown to confluence and transfected with siRNA and negative control for 24 h, respectively. To inhibit Hv1 activity, 1 M ZnCl solution was added into the DMEM medium to a final concentration of 100 µM [1], [2]. The SW620 and SW480 cells were planted in a 24-well plate (1.5×10^5^ per well) and cultured for 24vh to confluence and subsequently wounded with a tip. Cell movement was observed under phase-contrast microscopy, and were captured with a digital camera every 12 h.

### Invasion and migration assays

*In vitro* invasion and migration assays were performed to assess the effects of Hv1 on invasive and migratory abilities of SW620 and SW480 cells. SW620 and SW480 cells were cultured in six-well plate in DMEM medium with 10% FBS at confluence, transfected with the siRNA and negative control respectively for 24 h, and then trypsinized, washed and counted. For cell invasion, transwells with 8 µm pore size filters (Millipore) were covered with matrigel (Becton Dickinson) and inserted into 24-well plates. And for cell migration, the transwells were not coated with matrigel. DMEM medium (500 µl) containing 10% FBS was added to the lower chamber, and 200 µl of a cell suspension (5×10^4^ cells) was placed in the upper chamber. The plates were incubated at 37°C in humidified atmosphere containing 5% CO_2_ for 24 h. To inhibit Hv1 activity, 1 M ZnCl solution was added into the DMEM medium to a final concentration of 100 µM [1], [2]. The penetrated cells were fixed by paraformaldehyde, stained with crystal violet solution, and photographed. Each experiment was conducted four times. The migration and invasion rates were calculated as [migration cell No. of test/migration cell No. of control_SW620_] ×100% and [invasion cell No. of test/invasion cell No. of control_ SW620_] ×100%, respectively.

### Activity of Hv1 channel

The activity of Hv1 in colorectal cancer cells was assessed as a change in intracellular pH (pH_i_) in response to membrane depolarization by BCECF fluorescence [11]. The cells were incubated with 3.0 µM of BCECF-AM (Molecular Probe) in serum-free DMEM medium for 30 min, respectively, and washed with PSS solution (140 mM NaCl, 5 mM KCl, 5 mM glucose, 1 mM CaCl_2_, 1 mM MgCl_2_, 20 mM Tris, pH 7.5) for 3 times. Cells were incubated with NH_4_Cl/NMDG (N-methyl D-glucamine) solution (100 mM NMDG, 40 mM NH_4_Cl, 5 mM KCl, 5 mM glucose, 1 mM CaCl_2_, 1 mM MgCl_2_, 20 mM Tris, pH 7.3) for 20 min and washed by ammonium free solution (140 mM NMDG, 5 mM KCl, 5 mM glucose, 1 mM CaCl_2_, 1 mM MgCl_2_, 20 mM Tris, pH 7.4), rapidly inducing intracellular acidification [5]. Membrane depolarization was achieved by loading high K^+^ solution (145 mM KCl, 5 mM glucose, 1 mM CaCl_2_, 1 mM MgCl_2_, 20 mM Tris, pH 7.5). Intracellular pH changes acid-loaded cells were detected by fluorescent probe BCECF at excitation wavelengths of 490 nm and 440 nm and an emission wavelength of 525 nm under membrane depolarizing condition using RF-5301PC Spectrofluorophotometer (Shimadzu, Japan).

### Measurements of intracellular pH

Intracellular pH was measured using the pH sensitive fluorescent probe BCECF-AM. Cells cultured in the monolayers were incubated with 3.0 µM of BCECF-AM (Molecular Probe) in bicarbonate-free DMEM medium at 37°C for 30 min. After loading, the cells were washed three times with HEPES buffer to remove the extracellular dye, and made to remain in the identical buffer. The HEPES buffer contained 140 mM NaCl, 5 mM KCl, 1 mM MgSO_4_, 1 mM CaCl_2_, 1 mM NaH_2_PO_4_, 5.5 mM glucose, and 20 mM HEPES, pH 7.4. The fluorescence at excitation wavelengths of 490 nm and 440 nm was recorded at an emission wavelength of 525 nm using RF-5301PC Spectrofluorophotometer (Shimadzu, Japan). Calibration of fluorescence vs pH was performed by equilibration of external and internal pH with nigericin (10 µM) in a high K^+^ buffer with a range of pH from 5.5 to 8.0. The high K^+^ buffer contained 145 mM KCl, 5 mM glucose, 1 mM CaCl_2_, 1 mM MgCl_2_, and 20 mM HEPES (or MES). The relative fluorescence ratio values were plotted against corresponding pH_i_ values, which allowed determination of the unknown pH_i_.

### Statistical analysis

All statistics were performed using SPSS16.0 software. Measurement data was represented as mean ± SD. Comparison of the mean between groups was performed by *t* test. *P* values <0.05 were considered significant. Survival analysis was assessed using Kaplan-Meier method and survival rate was compared by log-rank test.

## Results

### Clinicopathologic findings

Clinicopathologic profiles from the 139 cases selected for this study were reviewed in Table 1. The average age of the patients was 62.4 years (range, 36–76), including 68 males and 71 females. The locations of their cancers were 29 left-sided and 43 right-sided colon, and 47 rectum cases, respectively. Among the 139 resected cases, the primary tumor size varied as follows: <5 cm in 72 cases, and ≥5 cm in 67 cases. Twenty of 139 tumors showed poor cytological differentiation. The tumor extent was limited (T1 or T2) in 45 cases and advanced (T3 or T4) in 94 cases. Tumor metastasis to the lymph nodes was observed in 59 of 139 cases.

### Increased expression of Hv1 in colorectal cancer

**Figure 1:**
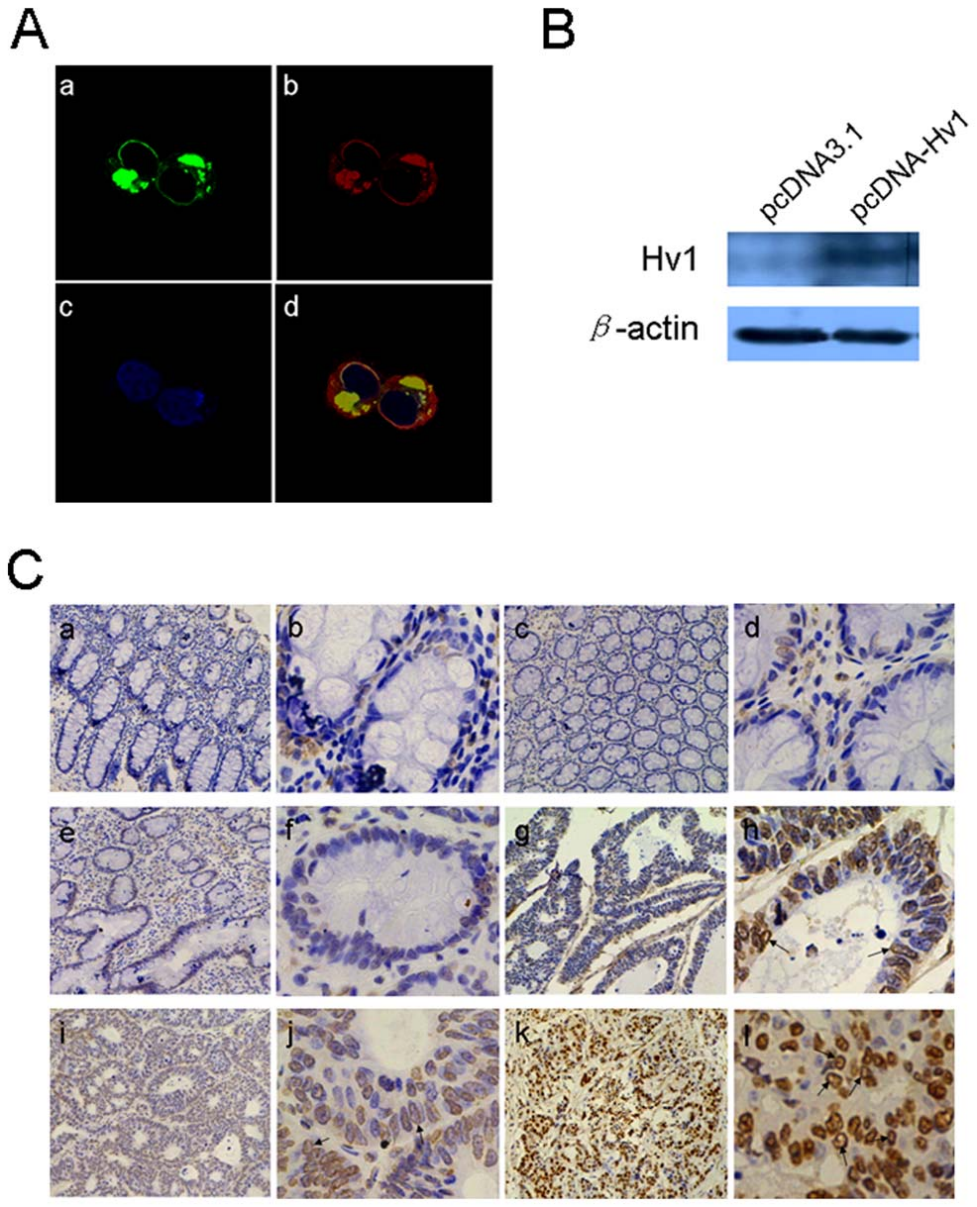
The anti-Hv1 antibody was used to detect recombinant Hv1 expressed in 293 T cells and native Hv1 protein in colorectal cancer tissues. A, 293 T cells were transfected with pHv1-EGFP plasmid and observed by a Leica TCS SP5 focal microscopy. a, observation by EGFP fluorescence. Hv1-EGFP fluorescence is represented in green. b, anti-Hv1 immunofluorescence (TRITC wavelengths) (red). c, DAPI stain to visualize the nuclei (blue). d, image merged is composite of EGFP fluorescence, anti-Hv1 immunofluorescence, and DAPI stain. B, 293 T cells were transfected with pcDNA3.1 vector and pcDNA-Hv1 plasmid, respectively. And Hv1 was detected by western blotting. C, Hv1 is expressed in colorectal cancer tissues. a (100×) and b (500×), c (100×) and d (500×), normal colorectal tissues; e (100×) and f (500×), adenoma tissues; g (100×) and h (500×), i (100×) and j (500×), k (100×) and l (500×), colorectal cancer tissues. In normal colorectal tissues, hyperplastic polyps and colorectal adenoma tissues, the staining was negative or weakly positive. In tumor tissues, intense immunoreactivity to Hv1 was observed. Hv1 is mainly observed in plasma membrane (h, j and l) (as indicated by arrowheads). The Hv1 is stained in brown, while the background nuclei are in blue.

Hv1 expressed in immune cells is associated with “respiratory burst” [3], [4], but its function in tumorigenesis has not been identified. To investigate Hv1 for use as a potential biomarker and therapeutic target for colorectal cancer, Hv1 expression in 139 colorectal cancer tissues and paired normal tissues, 10 normal colorectal, 20 colorectal adenoma and 18 hyperplastic polyp tissues, was detected using immunohistochemistry with an anti-Hv1 polyclonal antibody that was generated in house. To examine the specificity of the antibody, 293 T cells were transfected with pHv1-EGFP expression plasmid. And the expression of Hv1-EGFP was detected by immunocytochemistry and western blotting with the antibody, and EGFP fluorescence. The results showed that the antibody specifically recognizes Hv1 and EGFP is a marker for Hv1 expression (Fig. 1A and B).

As shown in Fig. 1C and Table 2, Hv1 staining was mainly moderate or strong positive in colorectal cancer tissues, but not in normal colorectal and hyperplastic polyp tissues. Hv1 was mainly observed in the plasma membrane of tumor cells in colorectal tissues, as shown in Fig. 1C (h, j and l) (as indicated by arrowheads). In colorectal adenoma tissues, the staining was negative or weakly positive (Fig. 1C, e and f; Table 2). Hv1 in colorectal cancer tissues was significantly expressed compared with that in normal colorectal, hyperplastic polyps and adenoma tissues, suggesting that Hv1 may be involved in colorectal tumorigenesis. Overall, 106 of the 139 (76.3%) cases showed high expression Hv1 in the tumor tissues (IRS over 3), while 33 (23.7%) of the cases showed low expression (IRS 0–3). Generally, Hv1 density was significantly higher in cancer tissues than in adenoma tissues (7.20±3.25 versus 2.20±2.12) (Table 2).

**Table 2:** Average density of immunohistochemistry staining.

Type of tissues	Staining density			*P*
	Mean	Standard deviation	Score range	
Normal colorectal	0.00	0.00	0	<0.001
Hyperplastic polyps	1.15	0.75	0–2	<0.001*
Adenoma	2.20	2.12	1–8	<0.001**
Colorectal cancer	7.20	3.25	1–12	<0.001***

* , 0.001; **, 0.001; and ***, 0.001 were compared with normal colorectal.

### High Hv1 expression is associated with a poor prognosis

The correlations between Hv1 expression and clinicopathologic characteristics are summarized in Table 3. There were significant associations with the depth of tumor classification (*P* = 0.007), age (*P* = 0.021), tumor size (*P* = 0.000), lymph node status (*P* = 0.000), clinical stage (*P* = 0.000) and p53 status (*P* = 0.014) in patients who had high Hv1 expression compared with patients who had low Hv1 expression. There was no significant association between Hv1 expression and the other clinical features, such as gender, differentiation and Ki-67 expression. In addition, the correlations between the expression levels of p53, Ki-67, TopoII, GST-π and P-gp and clinicopathologic characteristics were showed in Table 4. p53 status related to tumor classification (*P* = 0.011), Ki-67 to differentiation (*P* = 0.010), TopoII to differentiation (*P* = 0.010), and GST-π to tumor size (*P* = 0.007) and differentiation (*P* = 0.000). However, P-gp did not show statistical significance with clinicopathologic parameters.

**Table 3:** Correlation between Hv1 expression levels in colorectal cancer and clinicopathological parameters.

Characteristics		High	Low	*P*
		(n = 106, 76.3%)	(n = 33, 23.7%)	
Gender	Male	56(82.4)	12(17.6)	0.098
	Female	50(70.4)	21(29.6)	
Age (yr)	>62	66(83.5)	13(16.5)	0.021
	≤62	40(66.7)	20(33.3)	
Primary site	Left colon	25(86.2)	4(13.8)	0.207
	Right colon	34(79.1)	9(20.9)	
	Rectum	47(70.1)	20(29.9)	
Tumor size (cm)	<5	46(63.9)	26(36.1)	0.000
	≥5	60(89.6)	7(10.4)	
Tumor classification	T1/T2	28(62.2)	17(37.8)	0.007
	T3/T4	78(83.0)	16(17.0)	
Lymph node status	Negative	52(65.0)	28(35.0)	0.000
	Positive	54(91.5)	5(8.5)	
Distance metastasis	M0	99(75.0)	33(25.0)	0.130
	M1	7(100)	0(0.0)	
Clinical stage	I/II	50(64.1)	28(35.9)	0.000
	III/IV	56(91.8)	5(8.2)	
Differentiation	Well	45(73.8)	16(26.2)	0.589
	Moderately	44(75.9)	14(24.1)	
	Poorly	17(85.0)	3(15.0)	
p53 status	≤25%	48(67.6)	23(32.4)	0.014
	>25%	58(85.3)	10(14.7)	
Ki-67 status	≤25%	51(73.9)	18(26.1)	0.519
	>25%	55(78.6)	15(21.4)	
Topo II status	−/+	81(75.7)	26(24.3)	0.777
	++	25(78.1)	7(21.9)	
GST-π status	−/+	14(77.8)	4(22.2)	0.871
	++/+++	92(76.0)	29(24.0)	
P-gp status	−/+	35(76.1)	11(23.9)	0.973
	++/+++	71(76.3)	22(23.7)	

**Table 4:** Correlation of Hv1 expression levels in colon cancer with clinicopathological parameters, p53, Ki-67, TopoII, GST-π and P-gp expression levels.

		p53			Ki67			Topo II			GST-π			P-gp		
Characteristics		High	Low	*P*	High	Low	*P*	High	Low	*P*	High	Low	*P*	High	Low	*P*
Gender	Male	30(44.1)	38(55.9)	0.268	36(52.9)	32(47.1)	0.551	12(17.6)	56(82.4)	0.141	56(82.4)	12(17.6)	0.106	44(64.7)	24(35.3)	0.589
	Female	38(53.5)	33(46.5)		34(47.9)	37(52.1)		20(28.2)	51(71.8)		65(91.5)	6(8.5)		49(69.0)	22(31.0)	
Age (yr)	>62	42(53.2)	37(46.8)	0.251	43(54.4)	36(45.6)	0.271	18(22.8)	61(77.2)	0.939	70(88.6)	9(11.4)	0.530	53(67.1)	26(32.9)	0.958
	≤62	26(43.3)	34(56.7)		27(45.0)	33(55.0)		14(23.3)	46(76.7)		51(85.0)	9(15.0)		40(66.7)	20(33.3)	
Primary site	Left colon	15(51.7)	14(48.3)	0.415	13(44.8)	16(55.2)	0.768	6(20.7)	23(79.3)	0.146	26(89.7)	3(10.3)	0.720	17(58.6)	12(41.4)	0.491
	Right colon	24(55.8)	19(44.2)		23(53.5)	20(46.5)		6(14.0)	37(86.0)		36(83.7)	7(16.3)		31(72.1)	12(27.9)	
	Rectum	29(43.3)	38(56.7)		33(49.3)	34(50.7)		20(29.9)	47(70.1)		59(88.1)	8(11.9)		45(67.2)	22(32.8)	
Tumor size (cm)	<5	30(41.7)	42(58.3)	0.076	32(44.4)	40(55.6)	0.274	15(20.8)	57(79.2)	0.525	68(94.4)	4(5.6)	0.007	50(69.4)	22(30.6)	0.510
	≥5	38(56.7)	29(43.3)		36(53.7)	31(46.3)		17(25.4)	50(74.6)		53(79.1)	14(20.9)		43(64.2)	24(35.8)	
Tumor classification	T1/T2	15(33.3)	30(66.7)	0.011	21(46.7)	24(53.3)	0.547	11(24.4)	34(75.6)	0.783	37(82.2)	8(17.8)	0.241	29(64.4)	16(35.6)	0.670
	T3/T4	53(56.4)	41(43.6)		49(52.1)	45(47.9)		21(22.3)	73(77.7)		84(89.4)	10(10.6)		64(68.1)	30(31.9)	
Lymph node status	Negative	38(47.5)	42(52.5)	0.696	39(48.8)	41(51.2)	0.658	19(23.8)	61(76.2)	0.812	70(87.5)	10(12.5)	0.910	51(63.8)	29(36.2)	0.357
	Positive	30(50.8)	29(49.2)		31(52.5)	28(47.5)		13(22.0)	46(78.0)		52(88.1)	7(11.9)		42(71.2)	17(28.8)	
Distance metastasis	M0	65(49.2)	67(50.8)	0.742	66(50.0)	66(50.0)	0.713	30(22.7)	102(77.3)	0.720	116(87.9)	16(12.1)	0.207	89(67.4)	43(32.6)	0.573
	M1	3(42.9)	4(57.1)		4(57.1)	3(42.9)		2(28.6)	5(71.4)		5(71.4)	2(28.6)		4(57.1)	3(42.9)	
Clinical stage	I/II	38(48.7)	40(51.3)	0.957	38(48.7)	40(51.3)	0.662	19(24.4)	59(75.6)	0.672	68(87.2)	10(12.8)	0.959	50(64.1)	28(35.9)	0.427
	III/IV	30(49.2)	31(50.8)		32(52.5)	29(47.5)		13(21.3)	48(78.7)		53(86.9)	8(13.1)		43(70.5)	18(29.5)	
Differentiation	Well	27(44.3)	34(55.7)	0.458	24(39.3)	37(60.7)	0.010	9(14.8)	52(85.2)	0.025	52(85.2)	9(14.8)	0.000	36(59.0)	25(41.0)	0.212
	Moderately	32(55.2)	26(44.8)		38(65.5)	20(34.5)		20(34.5)	38(65.5)		51(87.9)	7(12.1)		42(72.4)	16(27.6)	
	Poorly	9(45.0)	11(55.0)		8(40.0)	12(60.0)		3(15.0)	17(85.0)		2(10.0)	18(90.0)		15(75.0)	5(25.0)	

For p53 and Ki-67, ≤25%, as a low expression; >25%, as a high expression. For TopoII, GST-π and P-gp, −/+, as a low expression; >+, as a high expression.

**Figure 2:**
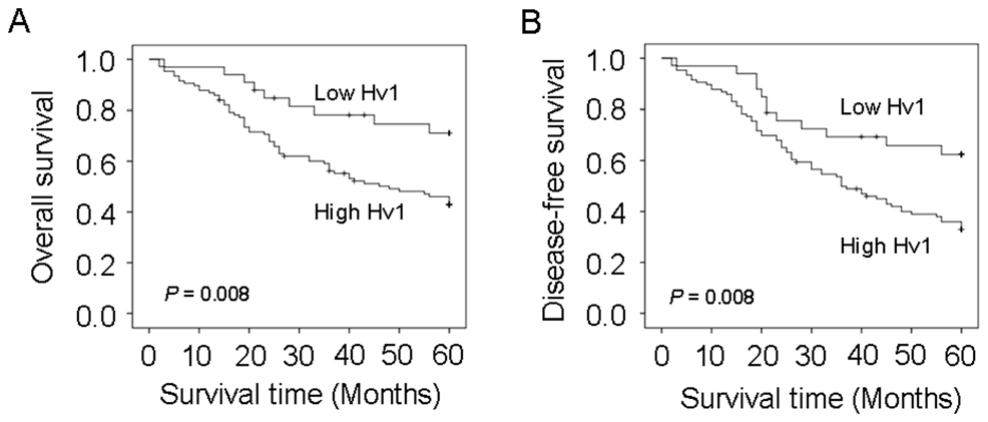
Cumulave overall and disease-free survival curves of patients with high and low expression of Hv1 in colorectal cancer tissues. The patients with low expression have longer overall and disease-free survival than the patients with high expression.

Kaplan-Meier survival curves showed that patients who had high Hv1 expression were more likely to have a shorter overall survival (*P* = 0.008, Fig. 2A) and recurrence-free survival (*P* = 0.008, Fig. 2B) compared with patients who had low Hv1 expression, suggesting that Hv1 over-expression may be associated with a poor clinical prognosis. Patients who had high Hv1 expression had a poor recurrence-free survival (*P* = 0.008) compared with patients who had low Hv1 expression (univariate analysis) (Table 5). Overall survival examined by Cox univariate analysis also indicated that high expression of Hv1 was significantly associated with shorter survival (*P* = 0.008). Univariate Cox regression analyses showed that Hv1 expression level was significantly associated with recurrence-free and overall survival, whereas other clinical characteristics lost their predictive significance. Furthermore, multivariate Cox regression analyses revealed that high expression of Hv1 was independent risk factor for overall survival (relative risk [RR] = 0.443, *P* = 0.015) and recurrence-free survival (RR = 0.427, *P* = 0.026) (Table 6). Patients who had high expression of Hv1 were prone to have an early recurrence compared with patients who had low expression of Hv1 (37.4±3.0 vs 47.2±10.7, *P*<0.008) (Table 5).

**Table 5:** Univariate logistic regression analysis of Hv1 expression.

Variable		Recurrence-Free Survival			Overall Survival		
		No. of Patients	Median (95% CI^a^)	*P*	No. of Patients	Median (95% CI^a^)	*P*
Hv1 expression	High	106	37.4(33.5–41.4)	0.008	106	40.0(35.9–44.1)	0.008
	Low	33	47.2(40.8–53.5)		33	50.7(45.0–56.5)	
Gender	Male	68	37.9 (33.2–42.7)	0.173	68	41.2(36.3–46.2)	0.414
	Female	71	41.5 (36.4–46.5)		71	43.8(38.8–48.8)	
Age (yr)	>62	79	37.5 (32.70–42.2)	0.302	79	38.6 (33.8–43.5)	0.024
	≤62	60	42.7 (37.7–47.6)		60	47.8 (43.0–52.5)	
Primary site	Left colon	29	38.1(30.3–45.8)	0.162	29	41.7(33.8–49.5)	0.193
	Right colon	43	34.7(28.3–41.1)		43	37.4(30.7–44.2)	
	Rectum	67	43.7(36.3–43.2)		67	46.2(41.4–50.9)	
Tumor size (cm)	<5	72	39.0(35.0–44.5)	0.91	72	42.8(38.0–47.6)	0.885
	≥5	67	39.7(34.7–44.7)		67	42.3(37.2–47.4)	
Differentiation	Well	61	41.0(35.9–46.2)	0.683	61	43.8(38.7–49.0)	0.653
	Moderately	58	38.9(33.5–44.3)		58	41.7(36.2–47.3)	
	Poorly	20	38.0(28.6–47.5)		20	40.9(31.0–50.7)	

a CI indicates confidence interval.

**Table 6:** Multivariate Cox proportional hazards analysis for recurrence-free survival and overall survival according to Hv1 expression.

Variable		Recurrence-Free Survival			Overall Survival		
		No. of Patients	RR^a^ (95% CI^a^)	*P* b	No. of Patients	Median (95% CI^a^)	*P* b
Hv1 expression	High	106	1.000	0.015	106	1.000	0.026
	Low	33	0.443(0.230–0.853)		33	0.427(0.202–0.903)	
Gender	Male	68	1.000	0.329	68	1.000	0.7
	Female	71	0.799(0.510–1.253)		71	0.908(0.554–1.486)	
Age (yr)	>62	79	1.000	0.329	79	1.000	0.104
	≤62	60	0.931(0.590–1.471)		60	0.650(0.387–1.092)	
Primary site	Left colon	29	1.000	0.329	29	1.000	0.421
	Right colon	43	1.225(0.690–2.175)		43	1.246(0.664–2.337)	
	Rectum	67	1.511(0.897–2.547)		67	1.461(0.824–2.591)	
Tumor size (cm)	<5	72	1.000	0.201	72	1.000	0.27
	≥5	67	0.730(0.451–1.182)		67	0.745(0.441–1.258)	
Differentiation	Well	61	1.000	0.685	61	1.000	0.752
	Moderately	58	0.761(0.386–1.500)		58	0.764(0.361–1.614)	
	Poorly	20	0.889(0.458–1.728)		20	0.877(0.421–1.826)	

a RR and CI indicate relative risk and confidence interval, respectively.

b *P* values were obtained by Cox proportional hazards analysis modeled for high and low/negative levels of Hv1 expression.

### Distribution of Hv1 in human colorectal cell lines

**Figure 3:**
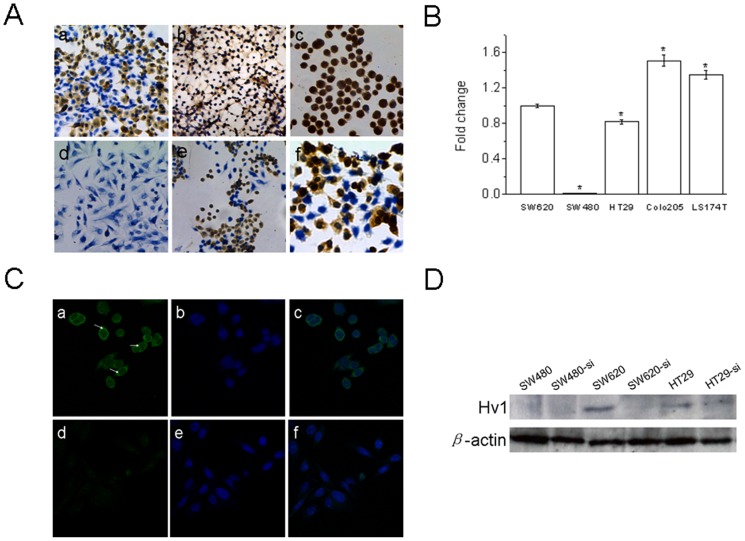
Expression of Hv1 in colorectal cancer cell lines, SW620, HT29, LS174T, Colo205 and SW480 cells. A, HT29 (a, 200×), LS174T (b, 200×), Colo205 (c, 200×), SW480 (d, 200×), SW620 (e, 200×) and SW620 (f, 500×) detected by immunocytochemistry. B, mRNA expression levels of Hv1 in SW620, HT29, LS174T, Colo205 and SW480 cells estimated by quantitative real-time PCR. Values are means ± SD (*n* = 3). * *P*<0.05, compared with SW620 cells. C, SW620 (a, b and c) and SW480 (d, e and f) cells grown on glass coverslips at confluence in a six-well tissue culture plate were stained with anti-Hv1 antibody and observed by a Leica TCS SP5 confocal microscopy. a and d, anti-Hv1 immunofluorescence (FITC, green). b and e, DAPI stain to visualize the nuclei (blue). c and f, images merged are composite of anti-Hv1 immunofluorescence and DAPI stain. Hv1 is observed in plasma membrane and intracellular sites in the highly metastatic SW620 cells (as indicated by arrowheads), but not in the poorly metastatic SW480 cells. D, expression of Hv1 in HT29, SW620 and SW480 cells, was detected by western blotting. Down-regulation of Hv1 expression was carried out by siRNA targeting Hv1 (HT29-si, SW620-si and SW480-si).

To examine whether Hv1 is also expressed in human colorectal cancer cell lines, the expression of Hv1 in human colorectal cancer cell lines, SW620, HT29, LS174T, Colo205 and SW480, was detected by immunocytochemistry and real time RT-PCR. As shown in Fig. 3, the expression levels of Hv1 among these colorectal cancer cell lines have significant difference. Hv1 is expressed at a high level in SW620, HT29, LS174T, and Colo205 cells, but not in SW480 cells. The localization of Hv1 in SW620 cells was determined by a confocal microscopy. As shown in Fig. 3C, Hv1 is highly expressed in SW620 cells, which is localized in both intracellular sites and plasma membrane (as indicated by arrowheads), whereas Hv1 is hardly expressed in SW480 cells. The result that Hv1 expression is higher in SW620 cells than that in SW480 cells is identical with the results from immunocytochemistry (Fig. 3A) and real time RT-PCR (Fig. 3B).

### Inhibition of Hv1 activity decreases invasion and migration in highly metastatic colorectal cancer cells

**Figure 4:**
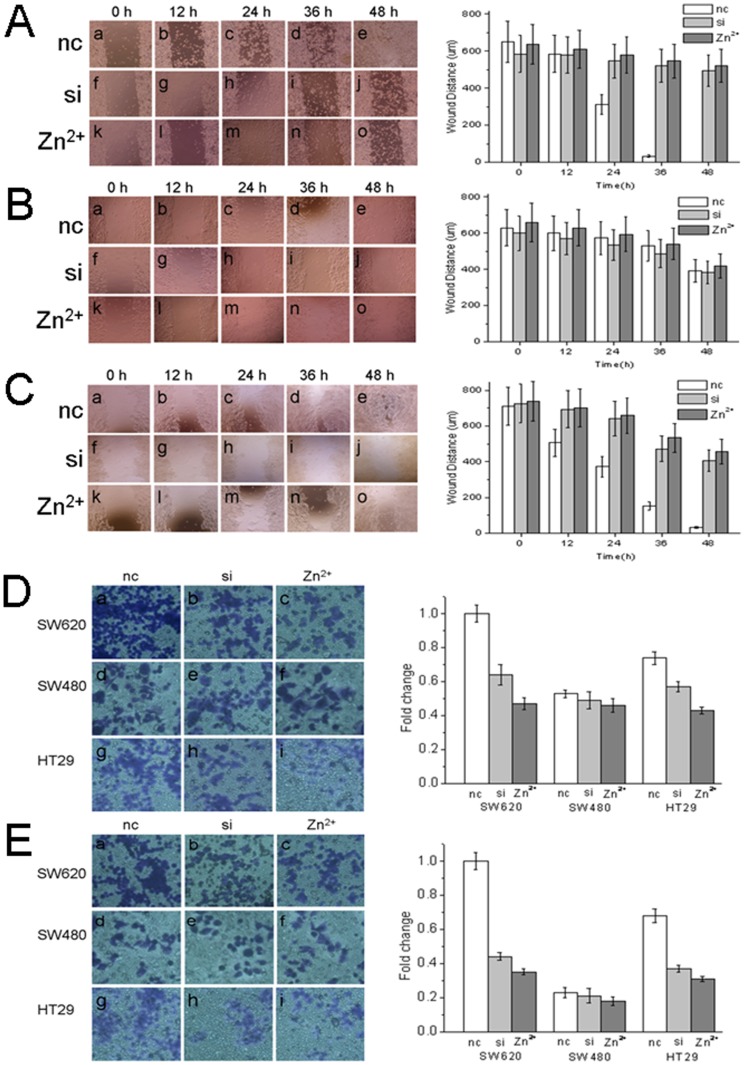
Hv1 increases colorectal cell migration and invasion. A, B and C, migration kinetics of SW620 (A), SW480 (B) and HT29 (C) assayed by wounded monolayer model. nc, cells transfected with the negative control (a, b, c, d and e in left panels); si, cells transfected with siRNA (f, g, h, i and j in left panels); and Zn^2+^, cells cultured at a final concentration of 100 µM ZnCl_2_ (k, l, m, n and o in left panels). Right panels in A, B and C show the time-dependent wound distances of SW620 (A), SW480 (B) and HT29 (C). Values are means±SD (*n* = 5). Migration of SW620 cells is faster than SW480 cells in wounded monolayers. Down-regulation Hv1 expression by siRNA or inhibition of Hv1 activity by 100 µM ZnCl_2_ clearly decrease the migratory ability of the highly metastatic SW620 cells. D and E, migration (D) and invasion (E) of SW620, SW480 and HT29 cells. Migration and invasion of the highly metastatic SW620 cells are significantly suppressed by down-regulation of Hv1 expression by siRNA targeting Hv1 and inhibition of Hv1 activity by 100 µM ZnCl_2_. Values are means ± SD (*n* = 3). *P*<0.05, compared with SW620 cells transfected with negative control.

Invasion and migration are two prominent hallmarks of tumor malignancy. To evaluate the contribution of Hv1 to invasive and migratory potential in colorectal cancer cells, we performed invasion and migration assays. First, we examined the kinetics of migratory ability of SW620, SW480 and HT29 cells. Fig. 4A, B and C showed the migration kinetics of SW620 (Fig. 4A), SW480 (Fig. 4B) and HT29 (Fig. 4C) cells. A wounded monolayer of SW620 cells allowed for wound closure after 48 h (Fig. 4A, a, b, c, d and e). SW620 and HT29 (Fig. 4C, a, b, c, d and e) cells closed the wound dramatically faster than SW480 cells (Fig. 4B, a, b, c, d and e). In order to down-regulation of Hv1 expression and inhibition of Hv1 activity, the siRNA targeting Hv1 and a final concentration of 100 µM ZnCl_2_ [1], [2] were used, respectively. The efficiencies of Hv1 knock-down with siRNA in SW620, SW480 and HT29 cells were assessed by western blotting, which showed the reduction in Hv1 protein levels upon siRNA knockdown in SW620 and HT29 cells (Fig. 3D). Suppressions of Hv1 expression by siRNA (f, g, h, i and j in Fig. 4A, B and C) and Hv1 activity by 100 µM ZnCl_2_ (k, l, m, n and o in Fig. 4A, B and C) clearly decreased the migration in SW620 and HT29 cells, but almost without affecting on SW480 cells (Fig. 4B, f, g, h, i and j for siRNA; k, l, m, n and o for 100 µM ZnCl_2_). The time-dependent wound distances of SW620 (Fig. 4A), SW480 (Fig. 4B) and HT29 (Fig. 4C) were shown in right panels.

We then studied invasion and migration of SW620, SW480 and HT29 cells using transwell inserts. As shown in Fig. 4D and E, SW620 cells have markedly higher invasive and migratory abilities than SW480 cells, which is consistent with the results from migration kinetics study above. Inhibitions of Hv1 expression by siRNA and Hv1 activity by ZnCl_2_ remarkably decreased the invasion and migration of SW620 cells, but almost did not influence SW480 cells (Fig. 4D and E). The data indicated that suppression of Hv1 expression and activity could inhibit the invasion and migration of the highly metastatic colorectal cancer cells *in vitro*, which suggested that Hv1 is involved in the invasion and migration of the metastatic human colorectal cancer cells.

### Hv1 involved in regulating intracellular pH

**Figure 5:**
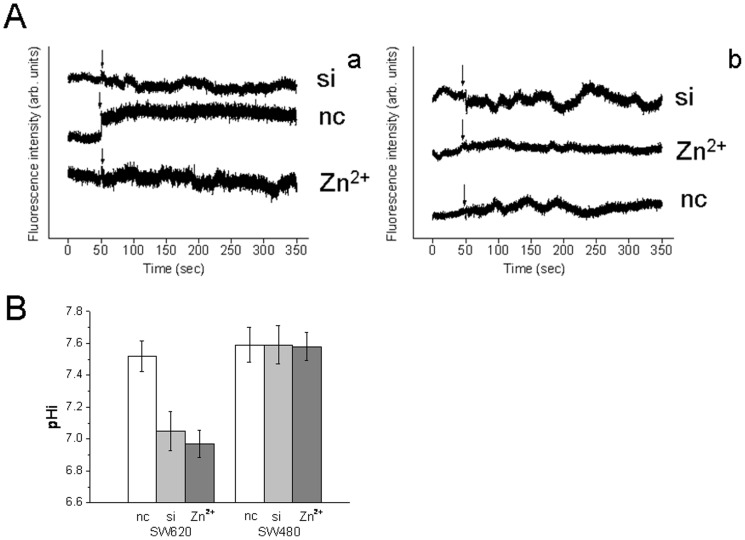
Hv1 regulates colorectal cancer cell intracellular pH. A, H^+^ channel activity of Hv1 in SW620 and SW480 cells. Membrane depolarization induces proton extrusion in SW620 cells (A, a (nc)), but does not affect in SW480 cells (A, b (nc)), whereas inhibition of Hv1 expression by siRNA (si) or 100 µM ZnCl_2_ (Zn^2+^) notably suppresses the proton extrusion in SW620 cells (A, a (si and Zn^2+^)). The arrowheads in A indicate the time when the high K^+^ solution was added into the culture dish, without interruptions in the recordings. B, Hv1 regulates intracellular pH of the highly metastatic colorectal cancer SW620 cells. Suppression of Hv1 expression by siRNA and activity by 100 µM ZnCl_2_ notably induces a decrease in intracellular pH in the highly metastatic SW620 cells.

The H^+^ channel activity of Hv1 in SW620 and SW480 cells was measured with a pH-sensitive probe BCECF. BCECF is a widely used pH indicator for estimating intracellular pH (pH_i_) [20], [21]. Its fluorescence intensity at maximum emission wavelength is pH-dependent: a fall in pH with a decrease in fluorescence intensity, and to a rise in pH with an increase in fluorescence intensity. We acid-preloaded and exposed SW620 and SW480 cells to an outward-acting proton force (in high-K^+^ medium) that will drive an efflux of H^+^ ions. As shown in Fig. 5A the sharp increase on the fluorescence intensity of BCECF at membrane depolarization was observed for SW620 cells (Fig. 5A, a (nc)), indicating that an increase in pH_i_ occurred. In contrast to SW620 cells, the florescence intensity of BCECF at membrane depolarization almost did not change for SW480 cells (Fig. 5A, b (nc)). Suppression of Hv1 expression obviously decreased the florescence intensity of BCECF at membrane depolarization in SW620 cells (Fig. 5A, a (si)), whereas did not affect on SW480 cells (Fig. 5A, b (si)). Inhibition of Hv1 activity by 100 µM ZnCl_2_ also inhibited outward proton extrusion in SW620 cells (Fig. 5A, a (Zn^2+^)). These results revealed that the pH_i_ recovery was due to active Hv1.

To examine the effect of Hv1 on intracellular pH (pH_i_), we measured the pH_i_ in SW620 and SW480 cells using BCECF. Down-regulation of Hv1 expression by siRNA and inhibition of Hv1 activity by 100 µM ZnCl_2_ induced a decrease in intracellular pH in the highly metastatic colorectal cancer SW620 cells, but not the poorly metastatic colorectal cancer SW480 cells (Fig. 5B). As shown in Fig. 5B, down-regulation of Hv1 expression in SW620 cells significantly increased acidity of intracellular pH from 7.5 to 7.0, while inhibition of Hv1 activity by 100 µM ZnCl_2_ more remarkably induced acidity of intracellular pH from 7.5 to 6.9 in SW620 cells. The finding showed that the inhibitions of Hv1 expression and activity in SW620 cells notably suppressed proton extrusion.

## Discussion

In the present study, our data revealed that Hv1 expression was markedly higher in colorectal cancer tissues than in normal colorectal tissues, colorectal adenoma tissues and colorectal hyperplastic polyp tissues. We observed that Hv1 expression in colorectal cancer was significantly associated with tumor recurrence and metastasis. Patients who had high expression of Hv1 were remarkably poor recurrence-free and overall survival compared with patients who had low expression of Hv1. Multivariate analysis demonstrated that Hv1 expression level was an independent prognostic factor for recurrence-free and overall survival in patients with colorectal cancer. Our results clearly demonstrated that high Hv1 expression is associated with poor prognosis and unfavorable clinical outcome of colorectal cancer.

To elucidate the mechanism that the effect of Hv1 on colorectal cancer development and metastasis, the expression of Hv1 in colorectal cancer cell lines was also detected, and the role of Hv1 in migration and invasion of colorectal cancer cells has been assessed. We found that Hv1 is highly expressed in highly metastatic colorectal cell lines, but lowly in poorly metastatic colorectal cell lines. Down-regulation of Hv1 expression or inhibition of Hv1 activity notably decreases the migratory and invasive abilities of the highly metastatic colorectal cancer cells. Suppression of Hv1 activity in the highly metastatic colorectal cancer cells restrains the extrusion of intracellular protons and induces the reduction of intracellular pH (pH_i_). Therefore, we conclude that Hv1 regulates intracellular pH (pH_i_) in the highly metastatic colorectal cancer cells.

Maintenance of cytosolic pH is vital for all biological processes in cells. Solid tumor cells often exist in a hypoxic microenvironment with an acidic extracellular pH (pH_o_) value than that of surrounding normal cells [22]–[24]. The high glycolytic activity and acidic metabolites in cancer cells result in the excessive production of intracellular acidity. To overcome the hypoxic microenvironment and prevent the intracellular accumulation of the increased acidic metabolites, tumor cells must be enhanced by the ability to dispose of the increased intracellular protons. Several pH_i_ regulatory mechanisms in tumor cells have been described, such as proton pumps, Na^+^/H^+^ exchangers, bicarbonate (HCO_3_^−^) transporters, and proton-lactate symporters, which have been shown to be involved in cancer progression and as promisingly therapeutic targets for future anticancer therapy [15], [25]–[31]. Recent researches have highlighted the fundamental role of the tumour's extracellular metabolic microenvironment in malignant invasion. This tumour cell microenvironment is acidified primarily by vacuolar H^+^-ATPases (V-ATPases) [15], Na^+^/H^+^ exchanger NHE1 [29], carbonic anhydrase [30] and H^+^/lactate cotransporter [31], which are activated in some cancer cells.

The importance of V-ATPases in cancer malignancy has been repeatedly demonstrated in several human cancer tumors and cell lines such as hepatocellular carcinoma [15], [32]. Inhibition of V-ATPase function *via* knockdown of the protein subunit ATP6L expression using RNA interfering technology can effectively retard the growth and metastasis of human hepatocellular carcinoma xenografts [15], [32]. Proton pump inhibitors (PPI), associated with the inhibition of V-ATPase activity and increasing in both extracellular pH and the pH of lysosomal organelles, trigger a rapid cancer cell apotosis in human melanomas, adenocarcinomas, lymphomas and B cells, as a result of intracellular acidification, caspase activation and early accumulation of reactive oxygen species in tumour cells [33]–[36]. Research groups in all over the world have recently started an International Society of Proton Dynamics in Cancer (ispdc) in January 2010 to investigate various aspects of proton dynamics in cancer cells [28]. The newly formed society contributes to stimulate interdisciplinary collaboration for the development of more specific and less toxic therapeutic strategies based on proton dynamics in tumor cell biology.

In our previous work, we showed that Hv1 function relates to breast tumor growth and metastasis through proton extrusion [17]. Hv1 is also expressed in airway epithelia, which mediates pH-regulated acid extrusion and acidify an alkaline airway surface liquid [37]. In the present work, the close relationship between Hv1 expression and clinicopathological features in colorectal cancer also predicted that Hv1 might boost carcinogenesis and tumor progression through regulating intracellular pH. Therefore, the voltage-gated proton channel Hv1 is a new candidate for some tumor cell intracellular and extracellular pH regulation.

The glycolysis and proton secretion in tumor cells are proposed to contribute to the proliferation and invasion of cancer cells during the process of tumorigenesis and metastasis [16], [38]. The cytosolic pH value is extremely important for tumor cells, inasmuch as a decrease of cytosolic pH possibly stops tumor cell metabolism and induces cell death [15], [33]. An alkaline cytosolic pH and an acidic extracellular pH resulting in high glycolytic activity and acidic metabolites are characteristics of tumor cells. The aberrant pH gradient between the alkaline cytosol and the acidic extracellular environment is involved in tumor progression and malignancy, which might be maintained by up-regulated activity of Hv1 that extrude protons outside the cell and acidify intracellular vesicles in colorectal cancer cells.

In conclusion, we demonstrated here that Hv1 is over-expressed in patients with colorectal cancer and high Hv1 expression correlated with the disease progression and poor clinical outcome in colorectal cancer. Furthermore, Hv1 was proved to be a risk factor for tumor recurrence and an independent molecular marker of prognosis for colorectal cancer and may become a novel molecular target in the strategies for the prediction of tumor recurrence and prognosis or treatment of colorectal cancer.

## References

[1] Ramsey IS, Moran MM, Chong JA, Clapham DE (2006) A voltage-gated proton-selective channel lacking the pore domain. Nature 440: 1213–1216. 16554753 10.1038/nature04700 PMC4084761

[2] Sasaki M, Takagi M, Okamura Y (2006) A voltage sensor-domain protein is a voltage-gated proton channel. Science 312: 589–592. 16556803 10.1126/science.1122352

[3] DeCoursey TE (2006) Voltage-gated proton channels and other proton transfer pathways. Physiol Rev 83: 475–579. 10.1152/physrev.00028.2002 12663866

[4] Henderson LM, Chappell JB, Jones OT (1987) The superoxide-generating NADPH oxidase of human neutrophils is electrogenic and associated with an H^+^ channel. Biochem J 246: 325–329. 2825632 10.1042/bj2460325 PMC1148280

[5] Clark RA, Leidal KG, Pearson DW, Nauseef WM (1987) NADPH oxidase of human neutrophils. Subcellular localization and characterization of an arachidonate-activatable superoxide-generating system. J Biol Chem 262: 4065–4074. 3031060

[6] Morgan D, Cherny VV, Murphy R, Katz BZ, DeCoursey TE (2005) The pH dependence of NADPH oxidase in human eosinophils. J Physiol 569: 419–431. 16195320 10.1113/jphysiol.2005.094748 PMC1464255

[7] DeCoursey TE, Cherny VV (1994) Voltage-activated hydrogen ion currents. J Membr Biol 141: 203–223. 7528804 10.1007/BF00235130

[8] Cherny VV, Markin VS, DeCoursey TE (1995) The voltage-activated hydrogen ion conductance in rat alveolar epithelial cells is determined by the pH gradient. J Gen Physiol 105: 861–896. 7561747 10.1085/jgp.105.6.861 PMC2216954

[9] Cherny VV, DeCoursey TE (1999) pH-dependent inhibition of voltage-gated H(+) currents in rat alveolar epithelial cells by Zn(2+) and other divalent cations. J Gen Physiol 114: 819–838. 10578017 10.1085/jgp.114.6.819 PMC2230650

[10] Long SB, Campbell EB, Mackinnon R (2005) Crystal structure of a mammalian voltage-dependent Shaker family K^+^ channel. Science 309: 897–903. 16002581 10.1126/science.1116269

[11] Koch HP, Kurokawa T, Okochi Y, Sasaki M, Okamura Y, et al (2008) Multimeric nature of voltage-gated proton channels. Proc Natl Acad Sci USA 105: 9111–9116. 18583477 10.1073/pnas.0801553105 PMC2449373

[12] Tombola F, Ulbrich MH, Isacoff EY (2008) The voltage-gated proton channel Hv1 has two pores, each controlled by one voltage sensor. Neuron 58: 546–556. 18498736 10.1016/j.neuron.2008.03.026 PMC2430592

[13] Lee SY, Letts JA, Mackinnon R (2008) Dimeric subunit stoichiometry of the human voltage-dependent proton channel Hv1. Proc Natl Acad Sci USA 105: 7692–7695. 18509058 10.1073/pnas.0803277105 PMC2409406

[14] Li SJ, Zhao Q, Zhou Q, Unno H, Zhai Y, et al (2010) The role and structure of the carboxyl-terminal domain of the human voltage-gated proton channel Hv1. J Biol Chem 285: 12047–12054. 20147290 10.1074/jbc.M109.040360 PMC2852942

[15] Fais S, De Milito A, You H, Qin W (2007) Targeting vacuolar H^+^-ATPases as a new strategy against cancer. Cancer Res 67: 10627–10630. 18006801 10.1158/0008-5472.CAN-07-1805

[16] Racker E (1972) Bioenergetics and the problem of tumor growth. Am Sci 60: 56–63. 4332766

[17] Wang Y, Li SJ, Pan J, Che Y, Yin J, et al (2011) Specific expression of the human voltage-gated proton channel Hv1 in highly metastatic breast cancer cells, promotes tumor progression and metastasis. Biochem Biophys Res Commun 412(2): 353–359. 21821008 10.1016/j.bbrc.2011.07.102

[18] Wang Y, Li SJ, Wu X, Che Y, Li Q (2012) Clinicopathological and biological significance of human voltage-gated proton channel Hv1 protein overexpression in breast cancer. J Biol Chem 87(17): 13877–13888. 10.1074/jbc.M112.345280 PMC3340163 22367212

[19] Li SJ, Zhao Q, Zhou Q, Zhai Y (2009) Expression, purification, crystallization and preliminary crystallographic study of the carboxyl-terminal domain of the human voltage-gated proton channel Hv1. Acta Crystallogr F65: 279–281. 10.1107/S1744309109003777 PMC2650464 19255483

[20] Chiche J, Ilc K, Laferrière J, Trottier E, Dayan F, et al (2009) Hypoxia-inducible carbonic anhydrase IX and XII promote tumor cell growth by counteracting acidosis through the regulation of the intracellular pH. Cancer Res 69: 358–368. 19118021 10.1158/0008-5472.CAN-08-2470

[21] Nilsson C, Kågedal K, Johansson U, Ollinger K (2003) Analysis of cytosolic and lysosomal pH in apoptotic cells by flow cytometry. Methods Cell Sci 25: 185–194. 15801164 10.1007/s11022-004-8228-3

[22] Griffiths JR (1991) Are cancer cells acidic? Br J Cancer 64: 425–427. 1911181 10.1038/bjc.1991.326 PMC1977628

[23] Gillies RJ, Liu Z, Bhujwalla Z (1994) 31P-MRS measurements of extracellular pH of tumors using 3-aminopropylphosphonate. Am J Physiol 267: C195–C203. 8048479 10.1152/ajpcell.1994.267.1.C195

[24] Raghunand N, Martínez-Zaguila′n R, Wright SH, Gillies RJ (1999) pH and drug resistance. II. Turnover of acidic vesicles and resistance to weakly basic chemotherapeutic drugs. Biochem Pharmacol 57: 1047–1058. 10796075 10.1016/s0006-2952(99)00021-0

[25] Gottlieb RA, Giesing HA, Zhu JY, Engler RL, Babior BM (1995) Cell acidification in apoptosis: granulocyte colony-stimulating factor delays programmed cell death in neutrophils by up-regulating the vacuolar H^+^-ATPase. Proc Natl Acad Sci USA 92: 5965–5968. 7541139 10.1073/pnas.92.13.5965 PMC41622

[26] Pouyssegur J, Sardet C, Franchi A, L′Allemain G, Paris S (1984) A specific mutation abolishing Na^+^/H^+^ antiport activity in hamster fibroblasts precludes growth at neutral and acidic pH. Proc Natl Acad Sci USA 81: 4833–4837. 6087349 10.1073/pnas.81.15.4833 PMC391585

[27] Gillies RJ, Martinez-Zaguilan R (1991) Regulation of intracellular pH in BALB/c-3T3 cells: bicarbonate raises pH via NaHCO_3_/HCl exchange and attenuates the activation of Na^+^/H^+^ exchange by serum. J Biol Chem 266: 1551–1556. 1846359

[28] Huber V, De Milito A, Harguindey S, Reshkin SJ, Wahl ML, et al (2010) Proton dynamics in cancer. J Transl Med 8: 57. 20550689 10.1186/1479-5876-8-57 PMC2905351

[29] Cardone RA, Casavola V, Reshkin SJ (2005) The role of disturbed pH dynamics and the Na^+^/H^+^ exchanger in metastasis. Nat Rev Cancer 5: 786–795. 16175178 10.1038/nrc1713

[30] Neri D, Supuran CT (2011) Interfering with pH regulation in tumours as a therapeutic strategy. Nat Rev Drug Discov 10: 767–777. 21921921 10.1038/nrd3554

[31] Parks SK, Chiche J, Pouyssegur J (2010) pH control mechanisms of tumor survival and growth. J Cell Physiol 226: 299–308. 10.1002/jcp.22400 20857482

[32] Lu X, Qin W, Li J, Tan N, Pan D, et al (2005) The growth and metastasis of human hepatocellular carcinoma xenografts are inhibited by small interfering RNA targeting to the subunit ATP6L of proton pump. Cancer Res 65: 6843–6849. 16061667 10.1158/0008-5472.CAN-04-3822

[33] Fais S (2010) Proton pump inhibitor-induced tumor cell death by inhibition of a detoxification mechanism. . J Intern Med. 267: 515–525. 20433578 10.1111/j.1365-2796.2010.02225.x

[34] Luciani F, Spada M, De Milito A, Molinari A, Rivoltini L, et al (2004) Effect of proton pump inhibitor pretreatment on resistance of solid tumors to cytotoxic drugs. J Natl Cancer Inst 96: 1702–1713. 15547183 10.1093/jnci/djh305

[35] De Milito A, Iessi E, Logozzi MA, Lozupone F, Spada M, et al (2007) Proton pump inhibitors induce apoptosis of human B cell tumors through a caspase-independent mechanism involving reactive oxygen species. Cancer Res 67: 5408–5417. 17545622 10.1158/0008-5472.CAN-06-4095

[36] De Milito A, Canese R, Marino ML, Borghi M, Iero M, et al (2010) pH-dependent antitumor activity of proton pump inhibitors against human melanoma is mediated by inhibition of tumor acidity. Int J Cancer 127: 207–219. 19876915 10.1002/ijc.25009

[37] Iovannisci D, Illek B, Fischer H (2010) Function of the HVCN1 proton channel in airway epithelia and a naturally occurring mutation, M91T. J Gen Physiol 136: 35–46. 20548053 10.1085/jgp.200910379 PMC2894549

[38] Gatenby RA, Gawlinski ET (2003) The glycolytic phenotype in carcinogenesis and tumor invasion: insights through mathematical models. Cancer Res 63: 3847–3854. 12873971

